# Encoding and estimation of first- and second-order binocular disparity in natural images

**DOI:** 10.1016/j.visres.2015.10.016

**Published:** 2016-03

**Authors:** Paul B. Hibbard, Ross Goutcher, David W. Hunter

**Affiliations:** a Department of Psychology, University of Essex, Colchester CO4 3SQ, UK; b School of Psychology and Neuroscience, University of St Andrews, St Mary’s Quad, South Street, St Andrews, KY16 9JP Scotland, UK; c Psychology, School of Natural Sciences, University of Stirling, Stirling FK9 4LA, Scotland, UK

**Keywords:** Binocular disparity, Depth perception, Second-order stereopsis, Natural images, Binocular energy model

## Abstract

The first stage of processing of binocular information in the visual cortex is performed by mechanisms that are bandpass-tuned for spatial frequency and orientation. Psychophysical and physiological evidence have also demonstrated the existence of second-order mechanisms in binocular processing, which can encode disparities that are not directly accessible to first-order mechanisms. We compared the responses of first- and second-order binocular filters to natural images. We found that the responses of the second-order mechanisms are to some extent correlated with the responses of the first-order mechanisms, and that they can contribute to increasing both the accuracy, and depth range, of binocular stereopsis.

## Highlights

• First- and second-order responses to natural binocular images are correlated.
• Second-order mechanisms can improve the accuracy of disparity estimation.
• Second-order mechanisms can extend the depth range of binocular stereopsis.

## 1 Introduction

### 1.1 The physiology of binocular vision

The binocular disparities provided by our front-facing eyes are an important cue to depth and distance. These disparities are encoded by binocular neurons in the visual cortex. These neurons, whose responses are affected by the images formed in both eyes, are found in many cortical areas, including V1 (Barlow et al., 1967, Hubel and Livingstone, 1987, Poggio and Poggio, 1984), V2 (Bredfeldt and Cumming, 2006, Thomas et al., 2002, Zhou et al., 2000), V3/V3A (Anzai et al., 2011, Cottereau et al., 2012) V4 (Shiozaki et al., 2012, Umeda et al., 2007) and V5/hMT+ (Krug & Parker, 2011). This wide spread of disparity sensitive areas across the cortex allows disparity processing to be specialised for distinct functional roles (Krug and Parker, 2011, Parker, 2007, Roe et al., 2007).

**Fig. 1:**
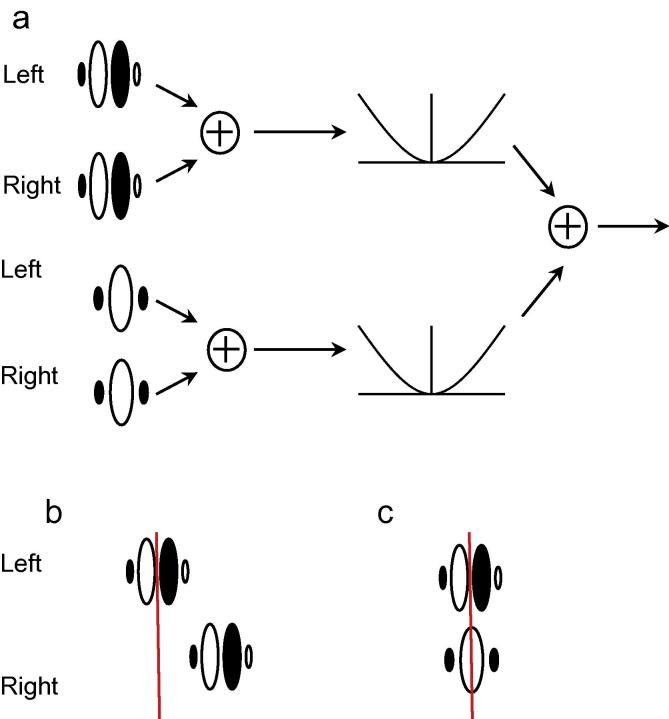
Outline of the binocular energy model. (a) Images are filtered with quadrature pairs of Gabor receptive fields. The white areas represent excitatory regions of the receptive fields, and the dark areas inhibitory regions. Responses are summed across the two eyes for corresponding filters, then squared. Finally, these squared outputs are summed across the two halves of the quadrature pair. (b) Positional disparity tuning is achieved if the two eyes’ receptive fields are in different locations. Here, the vertical red line shows the centre of the left eye’s receptive field; the right eye’s receptive field is identical in shape but shifted to the right. (c) Phase disparity tuning is achieved if the two eyes’ receptive fields have a different shape. Here, the two receptive fields are in the same location, but the left eye’s is odd-symmetric while the right eye’s is even symmetric.

The binocular energy model (DeAngelis et al., 1991, Fleet et al., 1996, Ohzawa et al., 1990) provides a good approximation to the responses of the initial stages of binocular processing in the primary visual cortex, although it does have some notable limitations (Faria, 2013, Haefner and Cumming, 2008, Read et al., 2002, Tanabe and Cumming, 2008). The basic structure of this model is outlined in Fig. 1. The first stage of the model consists of Gabor filters, in quadrature pairs, applied to each eye’s image. Binocular responses are created by first summing over corresponding pairs of filters in the two eyes, before squaring and summing again across the two components of the quadrature pair. For example, if the filters are in even-symmetric and odd-symmetric phase, the responses of the even filters to left and right eyes’ images are summed, as are the responses of the odd filters to the two images. The energy response is then created by squaring, then adding, these results.

Because the binocular energy model contains receptive fields in each eye, the overall response is affected by binocular disparities. This means that energy neurons can be created that are tuned for particular binocular disparities. In the case that the left and right eyes’ filters are identical, the model neuron will tend to respond best when the disparity is zero. Tuning to non-zero disparities can be achieved by introducing inter-ocular differences in the location or phase of the Gabor filters. Examples of these are shown in figure 1b and c, and both types of tuning are found in cortical neurons; typically, individual neurons will be tuned to non-zero disparities in both position and phase (Prince, Cumming, & Parker, 2002).

The binocular energy model is related to the concept of the use of cross-correlation as a way to match binocular images and estimate disparity (Allenmark, 2011, Banks et al., 2004, Fleet et al., 1996). In this model, the correlation between samples taken from the left and right images is calculated, at a range of disparities. High values of correlation can then be used as an indication of the similarity between the two samples, as a function of disparity, and thus used to estimate binocular disparity. This approach has been used to account for the spatial resolution of binocular depth (Banks et al., 2004) and the maximum gradient of disparity that supports binocular depth perception (Filippini & Banks, 2009). In order to approximate a cross-correlation on the basis of energy outputs, it is necessary to pool information across orientation, spatial frequency and space (Allenmark, 2011, Fleet et al., 1996) and to normalise responses to take account of differences in contrast between the samples from the two eyes (Hibbard, 2008). Thus, although the spatial limitations imposed by the initial binocular sampling in V1 can account for a number of limitations of depth perception (Banks et al., 2004, Filippini, 2009), the actual calculation of cross-correlation is likely to involve a more extended network of processing.

### 1.2 Depth perception from second-order binocular disparity

**Fig. 2:**
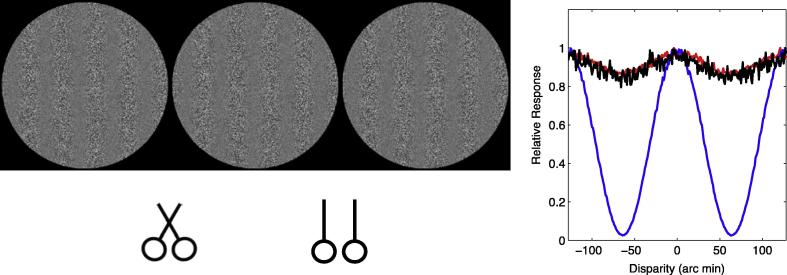
An example stimulus containing second-order disparity. The two eyes’ images consist of identical white noise samples that have been contrast modulated by a sinusoid. The phase of the sinusoid differs across the two images, creating a second-order disparity. The disparity in this stimulus is crossed, resulting in the perception of near depth. The left and centre images are arranged for crossed fusion, the centre and right images for uncrossed fusion. The graph shows the responses of first- and second-order mechanisms to this stimulus, as a function of the disparity in the contrast envelope. The blue line shows the response for a second-order mechanism tuned to a vertical orientation, and the same spatial frequency as the contrast envelope. The red and black lines show the responses of vertical first-order filters tuned to the same spatial frequency as the first- and second-stage filters of the second-order mechanism. Results are averaged over 500 sample images.

The first-stage filters of the binocular energy model are tuned to the orientation and frequency of the luminance variation in the monocular images. The disparity tuning of these model neurons means that they are sensitive to differences in the location of these luminance variations, which are referred to as first-order cues. In addition, depth can also be perceived on the basis of disparities in contrast-variations in this first-order information, even when there are no disparities in the first-order cues themselves (Hess and Wilcox, 1994, Wilcox and Hess, 1995, Wilcox and Hess, 1996, Wilcox and Hess, 1997, Schor et al., 1998, Edwards et al., 1999, Edwards et al., 2000, Langley et al., 1999, Langley et al., 1999, McKee et al., 2004). An example of these second-order, contrast-envelope cues is shown in Fig. 2. Here, the contrast of a Gaussian random noise sample (the first-order carrier) is modulated by a vertical sinewave (the second-order envelope). The noise carrier is identical for the left and right images, and presented at zero disparity. However, there is a non-zero (crossed) disparity in the modulation envelope. The fact that observers are able to perceive depth from contrast envelope cues in these stimuli, and others such as contrast modulated sinusoids, and Gabor patches in which the carrier and envelope disparity can be manipulated independently, has been used to argue for the existence of an additional, second-order stereoscopic channel (Hess and Wilcox, 1994, Wilcox and Hess, 1996, Langley et al., 1999, Langley et al., 1999).

### 1.3 Second-order mechanisms in the visual cortex

The perception of depth from disparities in contrast envelopes is an example of a more general phenomenon of second-order vision. This refers to situations in which perceived structure in the image cannot be derived from the outputs of linear filters at single points in the image (Graham & Sutter, 1998). In the case of the vertically-oriented contrast envelope in Fig. 2, for example, a linear filter tuned to the orientation and frequency of the envelope will not respond strongly and selectively to this stimulus.

The mechanisms that allow for the perception of this structure have variously been described as second-order, non-Fourier or complex (Graham, 2011, Graham and Sutter, 1998). Typically, they are modelled as two linear filtering stages, in which the responses of the first-stage filters are first transformed by a rectifying non-linearity, and secondly “paste[d] together” by the second-stage filters with larger receptive fields than those of the first-stage filters. (Graham & Sutter, 1998, p232).

Neurons in the prestriate cortex of both cats and monkeys have been found that are consistent with this filter-rectify-filter (FRF) model, and are therefore likely candidates as the physiological substrate of second-order channels. (Zhou and Baker, 1993, Zhou and Baker, 1994, Zhou and Baker, 1996, Rosenberg et al., 2010, Rosenberg and Issa, 2011; Tanaka & Ohzawa, 2006; Mareschal and Baker, 1998, Mareschal and Baker, 1998, Mareschal and Baker, 1999). These studies have identified neurons that respond to both first-order luminance-defined stimuli, and second-order, contrast-defined stimuli. These neurons have very similar tuning for orientation and spatial frequency in both luminance gratings, and the envelope of contrast-modulated gratings. In the cat, neurons have tended to be tuned to slightly higher luminance frequencies than envelope frequencies (Mareschal and Baker, 1998, Mareschal and Baker, 1999), while in the macaque monkey, this ratio is reversed (Li et al., 2014). For contrast modulated stimuli, these neurons are typically tuned for both the orientation and spatial frequency of both the carrier and envelope of the stimulus. In the FRF model, this tuning to the carrier reflects the properties of the early filter, and the tuning to the envelope the properties of the later filter. For contrast-modulated stimuli, the preferred carrier frequency reported was typically considerably higher than that of the contrast envelope. While there is a lot of variability across individual neurons, average estimates of the optimal spatial frequency ratio of 10 (Mareschal & Baker, 1999), 11.0 (Tanaka & Ohzawa, 2006) and 8.2 (Li et al., 2014) have been reported. There is also no clear relationship between the orientation tuning for the carrier and envelope (Mareschal and Baker, 1998, Mareschal and Baker, 1999), although Li et al. (2014) found a greater preponderance of neurons in which the carrier and envelope tunings were orthogonal.

The neurons described in these studies respond to both first-order and second-order stimuli. However, the methodology used to identify and record from cells typically does not allow for the detection of ‘pure’ second-order mechanisms, that do not respond to first-order structure, since the initial hunt for cells typically makes use of first-order stimuli (Schofield, Rock, Sun, Jiang, & Georgeson, 2010). Thus, while it is possible that pure second-order neurons might exist, empirical data have demonstrated the existence of neurons that respond to both first- and second-order stimuli.

### 1.4 The functional role of second-order mechanisms

A number of functional roles have been proposed for second-order mechanisms in vision. These include texture segmentation (Bergen & Landy, 1991), the ability to distinguish luminance changes arising from material changes from those arising from shape-from-shading (Schofield et al., 2006, Schofield et al., 2010), the perception of illusory contours (Shapley and Gordon, 1985, Wilson, 1993, Wilson and Richards, 1992) and the perception of transparency (Langley et al., 1999, Wilson et al., 1992).

One way to understand the role that second-order mechanisms might play is to analyse their responses to natural images, and to compare these with the responses of first-order filters. If the responses of second-order mechanisms are relatively uncorrelated with those of first-order mechanisms, this would suggest that the two provide complementary sources of information. Conversely, if the two are correlated, second-order mechanisms might still be beneficial in providing converging evidence for the presence of important image structures. For example, a potentially important role of second-order mechanisms is in segmenting images on the basis of texture differences (Bergen & Landy, 1991). Since boundaries between objects are often marked by changes in both texture and mean luminance, it is likely that first- and second-order mechanisms will provide consistent responses at the locations of boundaries. The pooling of first- and second-order information, as well as information from differences in other attributes such as colour, is beneficial in accurately locating object boundaries (Martin, Fowlkes, & Malik, 2004). This is because responses from multiple mechanisms are more likely to be correlated at true boundaries.

A number of studies have assessed the responses of second-order filtering operations to natural images. Schofield (2000) calculated the responses of second-order mechanisms to 8 natural images. The first stage of the model was a bank of linear filters, tuned to orientation and spatial frequency. The outputs of these were full-wave rectified, then used as the input for a bank of second-stage filters. Two versions of the model were used. In the first, specific-mapping model, the second-stage filters were always tuned to a frequency four octaves below the tuning of their first-order inputs, and responses were pooled across orientation prior to demodulation. The second, general-mapping model, did not pool first-stage filter outputs across orientation, and contained many mappings between first-stage filters and second-stage filters tuned to lower frequencies. Strong responses from both models were found at points in the images with high contrast, and in highly textured regions. Across images, no overall correlation was found between the raw pixel intensities in the image, and the second-order responses. This non-significant overall correlation reflects the fact that, for some images, there was a positive correlation, and for others a negative correlation.

Johnson and Baker (2004) also assessed the responses of second-order filters to natural images. They used an FRF model with full-wave rectification, and calculated responses for many combinations of orientation and spatial frequency tuning in the first- and second-order filtering stages. They found self-similar second-order structure in natural images, similar to that found for first-order information (Burton and Moorhead, 1987, Field, 1987, Field and Brady, 1997, Tolhurst et al., 1992, van der Schaaf and van Hateren, 1996). They calculated the correlation between second-order responses, and full-wave rectified first-order responses, and found an overall significant correlation. When they considered filters tuned to specific frequencies, the greatest correlations occurred when the second-stage filters were tuned to a frequency between 2 and 16 times lower than the frequency tuning of simple first-order filters. They also found stronger correlations between first- and second-order filters tuned to the same orientation. Johnson, Kingdom, and Baker (2005) also found significant unsigned correlations between the responses of a second-order luminance channel, and first-order channels tuned to both luminance and colour variations. Unsigned correlations between first-order channels and second-order colour channels were less pronounced.

These significant correlations for unsigned filter outputs are consistent with the overall lack of correlation found by Schofield (2000), if local correlations are sometimes positive and sometimes negative. This was assessed further by Schofield and colleagues (Schofield et al., 2010, Schofield et al., 2006), who proposed that the specific relationship between first- and second-order information can be used to distinguish luminance changes that result from shape-from-shading, from those that result from material changes. They showed that, while in-phase combinations of first- and second-order information tend to be perceived as shaded, corrugated surfaces, anti-phase combinations tend to be perceived as flat.

### 1.5 Second-order mechanisms for the encoding and estimation of binocular disparity

**Fig. 3:**
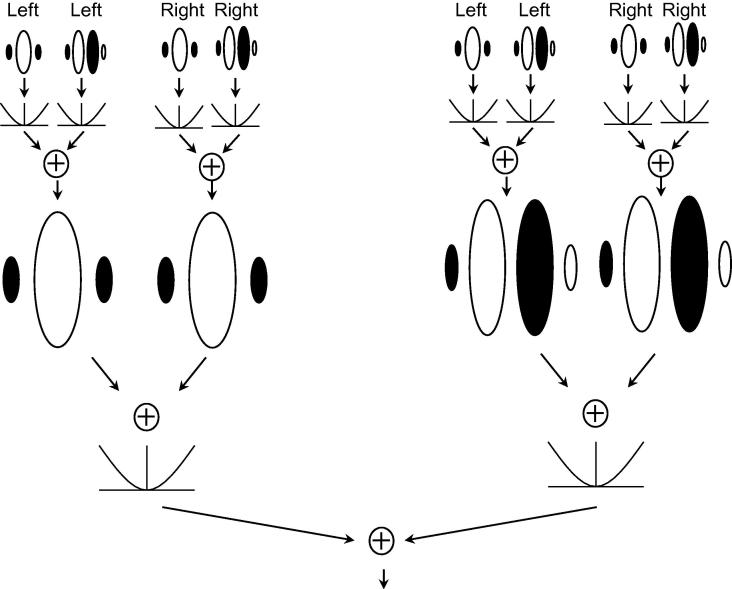
A second-order binocular energy model. Energy responses are first calculated separately for each eye. These monocular energy responses form the input to second-order filters, at a lower spatial frequency. These are then used to calculate a second-order binocular energy response in the same way as the standard first-order energy response.

Tanaka and Ohzawa (2006) showed that a subset of cortical neurons in cats respond to second-order binocular disparity cues. They modelled these second-order neurons using a filter-rectify-filter model (Zhou & Baker, 1993). The first stage of this model is the calculation of monocular energy, based on the outputs of standard linear Gabor filters, tuned to orientation and spatial frequency (Langley et al., 1996, Mareschal and Baker, 1998, Mareschal and Baker, 1998). The monocular energy outputs are then filtered again, using Gabor filters tuned to a much lower spatial frequency than the first-stage filters. A second-order binocular energy response is then calculated on the basis of the outputs of these second-stage filters. Our implementation of this second-order pathway is illustrated in Fig. 3. In this model, binocular combination only occurs at the final stage of the calculation of the second-order binocular energy response. Alongside this model, Tanaka and Ohzawa (2006) considered another model, in which binocular combination occurs before the second-stage filtering. However, the second-stage convergence model, which we have used here, provided a better account of their results, and is consistent with psychophysical results (Wilcox & Hess, 1996).

Although binocular neurons have a preferred disparity, they can nevertheless respond strongly to stimuli with non-preferred disparities. Indeed, across a population, responses from neurons tuned to incorrect disparities can be stronger than responses from neurons tuned to the correct disparity. This makes disparity estimation a difficult computational problem. One way in which this problem can be reduced is to pool information across neurons tuned to different orientations and frequencies, and across local spatial regions. Fleet et al. (1996) argued that this would help to reduce false peaks in the response, since large responses at the true disparity would tend to sum, while responses at incorrect disparities would not tend to occur in the same place across different scales, orientations and positions. Other pooling approaches, such as coarse-to-fine analysis (Chen & Qian, 2004) and the use of phase-tuned neurons to veto incorrect matches (Read & Cumming, 2007) have also been proposed. Tanaka and Ohzawa (2006) argued that such a pooling strategy might usefully be extended to include a second-order channel. They argued that this channel would capture information at a coarse scale, and that this might therefore form a useful component of coarse-to-fine or multichannel pooling approaches.

Wilcox and Hess (1997) argued that second-order mechanisms might provide a useful ‘back-up’ system for the coarse analysis of the global disparity of objects and surfaces. They proposed that first-order mechanisms are used when the matching problem is simple, and there is little matching ambiguity. In contrast, the second-order system might be useful in complex stimuli in which first-order matches are ambiguous, or simply do not provide a depth signal. Wilcox and Hess (1997) also argued that a second-order channel has the potential to increase the range of disparities that may be encoded within each spatial frequency band of the image. Psychophysical and physiological evidence suggests that there is a correlation between the size of the receptive fields of mechanisms underlying disparity processing, and the size of disparity to which they are tuned (Allenmark, 2011, McKee and Verghese, 2002, Prince et al., 2002, Smallman and MacLeod, 1994, Tsirlin and Allison, 2008, Tyler, 1973, Tyler, 1974, Tyler, 1975). When we consider the initial stage of bandpass filtering in the estimation of disparity (Goutcher and Hibbard, 2014, Harris, 2014), a link between the spatial frequency tuning of filters and the range of disparity tuning is also expected. For a given bandwidth of filter, the size of the receptive field is inversely proportional to its frequency tuning. It is possible to increase the spatial area sampled by decreasing the spatial frequency bandwidth of the filter, or by pooling over multiple filters within a local spatial region (Fleet et al., 1996). Second-order filters provide a mechanism for this local spatial pooling. These second-order filters are tuned to much lower spatial frequencies than their first-stage linear filters (Tanaka & Ohzawa, 2006). This allows for the possibility of a broader range of disparity tuning, even to information at the spatial frequency of relatively high carrier frequencies.

This link between the second-order channel and the perception of relatively large disparities has been found in psychophysical studies. Wilcox and Hess (1995) measured the upper limit for the perception of disparity in Gabor stimuli, and found that it was determined purely by second-order information. As the envelope size increased, the upper disparity limit increased, regardless of the spatial frequency of the carrier. This allowed for the perception of disparities of many multiples of the carrier frequency. Such disparities are a difficulty for mechanisms based on the outputs of bandpass filters, since these are quasi-periodic and thus provide multiple false candidates for disparity matching, at a separation determined by the frequency tuning of the filter.

The spatial pooling, and low spatial frequency tuning, of second-order disparity mechanisms (Tanaka & Ohzawa, 2006) therefore suggest that they may be able to improve the accuracy of disparity detection, and increase the range of disparities detected at a given spatial frequency.

### 1.6 Goals of the current study

In the current study, we compared the first- and second-order information available in natural binocular images, and assessed the extent to which second-order filters can improve disparity estimation relative to a model based purely on first-order filters. To do this, we first calculated the phase disparity information in the responses of our first- and second-order channels to a collection of natural binocular images. This allowed us to determine the extent to which first- and second-order disparity signals are correlated in binocular natural images, and conversely the extent to which the second-order channel provides information that is not available in the first-order channel. To determine whether the second-order channel can improve the accuracy of disparity estimation in natural images, we examined the distributions of disparity estimates derived from first- and second-order channels to naturalistic images, with a constant, known, disparity. To do this, rather than as in the previous analysis, we presented the left half-image from each binocular pair to both eyes, at a fixed disparity. This technique is similar to that used by Okajima (2004) and Burge and Geisler (2014). In this way, we simplified the disparity estimation problem, and provided stimuli with a known disparity. Unlike many artificial stimuli such as random dot stereograms, and Gabor patches, our stimuli therefore had the luminance statistics of natural images.

## 2 Method

### 2.1 The image data set

The methods for capturing and processing the binocular images are described in Hibbard (2008), and summarised here for convenience. Images were captured using two Nikon Coolpix 4500 digital cameras, harnessed in a purpose-built mount, with an inter-camera separation of 65 mm. The cameras were oriented so that the same point in the scene projected to the centre of each camera’s image.

**Fig. 4:**
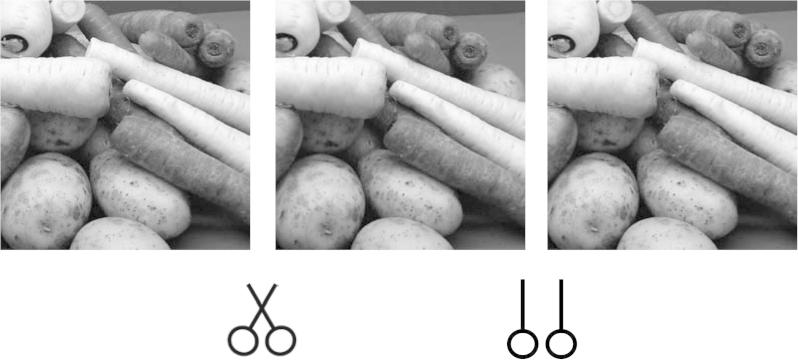
An example of the binocular stimuli used. The left and centre images are arranged for crossed fusion, then centre and right images for uncrossed fusion.

Two classes of scene were investigated. In the first, images were collections of natural objects (fruit, vegetables, stones, shells, plants) arranged in ‘still-life’ collections. The second collection was of outdoor scenes. Since the cameras were fixated on a target object in each image pair, and a range of distances was sampled, the images contain a range of convergence distances, from approximately 50 cm to tens of metres. 139 binocular image pairs were analysed. An example image pair is shown in Fig. 4.

Images were captured at a resolution of 1600 × 1200 pixels. They were then calibrated to take account of the characteristics of the cameras. Firstly, images were calibrated to correct for lens distortions, calculate the effective focal lengths of the cameras, and transform the images into a ‘pinhole-camera’ model. The final resolution of the images was 1 pixel per arc minute of visual angle. The images were also calibrated to take account of the colour characteristics of the cameras, by capturing colour patches from a Macbeth Colorchecker DC chart, and using these to map RGB camera values to CIE LAB values (Hong, Luo, & Rhodes, 2001). Analyses were performed on the luminance information only.

### 2.2 The binocular energy model

Our implementation of the binocular energy model is that described in detail by Hibbard (2008). In summary, the first stage of filters are defined as:

$$G_{L\text{,}R}\left(x\text{,}y\text{;}f\text{,}\theta \text{,}\sigma \text{,}\eta \text{,}x_{L\text{,}R}\text{,}y_{L\text{,}R}\right)=e^{\left(\frac{-{\left(\overset{´}{x}-x_{L\text{,}R}\right)}^{2}}{2\sigma^{2}}\frac{-{\left(\overset{´}{y}-y_{L\text{,}R}\right)}^{2}}{2\eta^{2}}\right)}\cdot [\cos\left(2\pi f\left(\overset{´}{x}-x_{L\text{,}R}\right)\right)+i\sin\left(2\pi f\left(\overset{´}{x}-x_{L\text{,}R}\right)\right)]$$

where

(1) $$\left[\begin{array}{c} \overset{´}{x}\\ \overset{´}{y} \end{array}\right]=\left[\begin{array}{cc} \cos\theta & -\sin\theta \\ \sin\theta & \cos\theta \end{array}\right]\cdot \left[\begin{array}{c} x\\ y \end{array}\right]$$

where *L*,*R* refer to filters that respond to the left and right eye’s images, respectively. Responses to the left and right eye’s images are given by its convolution with the image:

(2) $$R_{L\text{,}R}\left(x\text{,}y\right)=G_{L\text{,}R}\left(x\text{,}y\right)*I_{L\text{,}R}\left(x\text{,}y\right)$$

The binocular energy response is then given by:

(3) $$E_{B}={\left|R_{L}\left(x\text{,}y\right)+R_{R}\left(x\text{,}y\right)\right|}^{2}$$

The energy model is illustrated in Fig. 1. The standard deviations of the Gaussian envelope, *σ* and *η* were set at $\frac{0.39}{f}$ and $\frac{0.78}{f}$ arc min, respectively.

### 2.3 Second-order energy model

The architecture of the second-order model, shown in Fig. 3, was based on the second-stage convergence model proposed by Tanaka and Ohzawa (2006). In this model, first-stage filtering is followed by a monocular energy calculation (i.e. the summation of pairs of monocular filters in quadrature phase, and the squaring of the result), followed by second-stage filtering. Binocular combination occurs only at a final stage of binocular energy calculation based on the outputs of the second-stage filters.

### 2.4 Spatial frequency and orientation tuning of the filters

Physiological studies report a wide variety of relationships between the spatial frequency and orientation tuning of the first and second stages of filtering for neurons that respond to second-order stimuli. To capture this, we created second-order filters in which the second-stage filters were tuned to 0.2 or 0.4 cpd and the first-stage filters were tuned to a frequency that was 5, 10 or 20 times higher. In all cases, the orientation of the second-stage filter was vertical, to capture horizontal disparities, and the first-stage filters were oriented at 0, ±45 or 90 degrees from horizontal. This resulted in 12 varieties of filter for each second-order spatial frequency. The range of frequency ratios used covers a similar range to that found in physiology (Tanaka & Ohzawa, 2006) for binocular second-order mechanisms.

The responses of the second-order mechanisms were compared with the responses of vertically tuned first-order mechanisms tuned to both the first- and second-stage filters. This required the filtering with mechanisms tuned to frequencies of 0.2, 0.4, 1, 2, 4, and 8 cycles/degree.

### 2.5 Model responses to second-order stimuli

To demonstrate how the model responds to first- and second-order disparities, we calculated its responses to the contrast modulated noise stimulus shown in Fig. 2. A Gaussian white noise sample was contrast modulated by a 0.47 cpd grating with a modulation depth of 0.5. The disparity of the noise itself was kept at zero, while the disparity in the contrast modulation was varied between ±1 wavelengths (±128 arc min). We calculated the binocular energy response of a second-order mechanism with a vertical 4.7 cpd first-stage filter and a vertical 0.47 cpd second-stage filter, tuned to zero position and phase disparity. We also calculated the energy response for first-order channels tuned to the orientation and frequency of the first- and second-stage filters. For each mechanism, we calculated the mean energy response over 500 samples.

### 2.6 Binocular disparity information available in first- and second-order channels

Fleet et al. (1996) showed that the binocular energy response can be described in terms of the monocular amplitude and phase signals as follows:

(4) $$E_{B}^{}\left(x\text{,}y\right)=\rho_{L}^{2}\left(x\text{,}y\right)+\rho_{R}^{2}\left(x\text{,}y\right)+2\rho_{L}\left(x\text{,}y\right)\rho_{L}\left(x\text{,}y\right)\cos\left(\Delta \phi \left(x\text{,}y\right)\right)$$

where *Δϕ*(*x*,*y*) is the phase difference between the left and right signals:

(5) $$\Delta \phi \left(x\text{,}y\right)=\phi_{L}\left(x\text{,}y\right)-\phi_{R}\left(x\text{,}y\right)$$

and the monocular amplitude and phase are defined as:

(6) $$\phi_{L\text{,}R}\left(x\text{,}y\right)=\tan^{-1}\left(\frac{I\left[R_{L\text{,}R}\left(x\text{,}y\right)\right]}{R\left[R_{L\text{,}R}\left(x\text{,}y\right)\right]}\right)$$

and

(7) $$\rho_{L\text{,}R}\left(x\text{,}y\right)=\sqrt{R{\left[R_{L\text{,}R}\left(x\text{,}y\right)\right]}^{2}+I{\left[R_{L\text{,}R}\left(x\text{,}y\right)\right]}^{2}}$$

respectively, where R and I are the real and imaginary parts of the response. Thus, the binocular energy response consists of the sum of the two monocular responses, and a term that is modulated by the interocular phase difference. As phase is equivalent to a shift in the sinusoidal function, this phase difference is closely linked to the position disparity (Fleet et al., 1996). Image disparities between plus and minus half the wavelength of the sinusoid are linearly proportional to the phase disparity. Provided the image disparities are within the appropriate range, conversion from image to phase disparity is simply:

(8) $$\Delta I_{L\text{,}R}\left(x\text{,}y\right)f=2\pi \Delta \phi \left(x\text{,}y\right)$$

where $\Delta I_{L\text{,}R}\left(x\text{,}y\right)$ is the shift caused by image disparity between the left and right images.

We calculated the phase disparity in our first- and second-order channels as a measure of the binocular disparity information available in the outputs of the binocular energy mechanisms. Phase disparity was calculated by simply taking the difference between the phase of the response for the two eyes.

### 2.7 Disparity estimation

We were interested in the extent to which the second-order mechanisms could improve the accuracy of disparity estimation in natural images. To do this, we made two comparisons with estimates derived from first-order filters. These two comparisons reflect the fact that the second-order mechanisms consist of two filtering stages.

In the first analysis, we were interested in the extent to which the spatial pooling of the outputs of the high-frequency first-stage filters improved performance over that possible from these first-stage filters alone. This analysis therefore assessed the benefits accrued from the spatial pooling performed by the second-order mechanisms. In this case, one potential benefit of the second-order mechanism is to extend the range of disparities that can be detected. Since there is a correlation between the size of the receptive fields of mechanisms underlying disparity processing, and the size of disparity to which they are tuned (Allenmark, 2011, McKee and Verghese, 2002, Prince et al., 2002, Smallman and MacLeod, 1994, Tsirlin and Allison, 2008, Tyler, 1973, Tyler, 1974, Tyler, 1975), the first-stage filters will be relatively limited in the range of disparities to which they are tuned. One benefit of the second-stage filtering, which is tuned to a much lower frequency, would be to extend this range (Wilcox & Hess, 1997).

In the second analysis, we assessed the benefit of using a second-order mechanism, in comparison with a simple, first-order filter tuned to the same frequency as the second-stage filters. These filters can contribute directly to the detection of large disparities without the need for second-order mechanisms. However, these filters are tuned to low frequencies. A potential benefit of second-order mechanisms is therefore to support the detection of large disparities (due to the low-frequency tuning of the second-stage filters), but to use the high-frequency information available to the first-stage filters to do this.

Another possible benefit of second-order mechanisms is that their responses can be pooled with those of first-stage mechanisms in the estimation of disparity. Tanaka and Ohzawa (2006) argued that this pooling could be combined with the pooling of information over spatial position, orientation and spatial frequency in order to facilitate the rejection of false matches in solving the binocular correspondence problem (Fleet et al., 1996, Qian and Zhu, 1997). We assessed the potential benefit of pooling information across first- and second-order mechanisms in two ways. In the first analysis, we pooled the responses of the first- and second-order mechanisms, separately for each of the 12 combinations of first-stage orientation and spatial frequency tuning. Second-order responses were pooled with first-order responses that were tuned to the spatial frequency of the second-stage filters.

The existence of multiple second-order mechanisms, with differing first-stage orientation and frequency tuning properties for each of the second-stage filters also provides the opportunity for information from a broad range of high-frequency information to contribute to the estimation of disparity. We therefore performed an additional analysis. Rather than pooling the response of a single second-order mechanism with that of the first-order mechanism, we pooled across all 12 combinations of first-stage filters.

Binocular energy responses were calculated across a range of position-disparity tuned model neurons. Responses were calculated for 1000 locations randomly and uniformly sampled from each of the 139 images, giving a total of 139,000 sample locations. To create an idealised situation, we presented the same image to each eye, with fixed horizontal disparity. The estimated disparity was taken as the disparity tuning of the unit giving the largest response in each case (Chen & Qian, 2004). Mechanisms were tuned to ±1 wavelength of the filter, at intervals of 1 arc min. In the first analysis, disparities of 0, 5, 10 and 15 arc min were used. In the second analysis, disparities of 0, 15, 30, 45 and 60 arc min were used.

## 3 Results

### 3.1 Reponses to contrast modulated stimuli

Fig. 2 shows the binocular energy responses to contrast modulated noise stimuli, for a second-order mechanism and first-order mechanisms tuned to the orientation or frequency of the first-stage or second-stage filters. Responses are separately normalised against the maximum response. The second-stage filter is strongly modulated by the envelope disparity.

### 3.2 Distributions of phase disparities

**Fig. 5:**
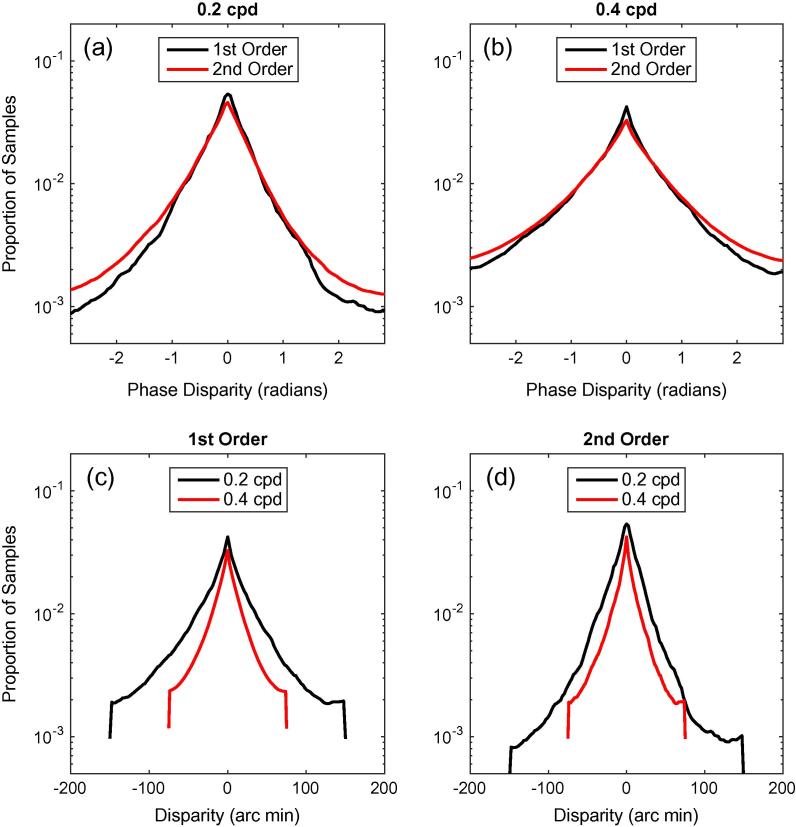
(a and b) Distributions of phase disparities in the responses of first- and second-order mechanisms for spatial frequency tunings of (a) 0.2 cpd and (b) 0.4 cpd. The first-order results are for a vertically-oriented filter. The second-order results are for a mechanism with a vertical second-stage filter, and are pooled over all 12 combinations of frequency ratio and orientation in the first-stage filters. (c) These distributions are replotted as a function of the equivalent positional disparity, for the two frequencies of first-order filters. (d) Results plotted in the same way for the second-order mechanisms.

Fig. 5 shows the distributions of phase disparities in the responses of the Gabor filters. Fig. 5a shows the responses of the first- and second-order channels, tuned to vertical image structure, with both channels tuned to 0.2 cpd. In all cases, the histograms show the distributions of phase disparities from all image locations in all of the binocular image pairs tested. For the second-order channel, the responses of all 12 combinations of orientation and spatial frequency in the first-stage filters, are pooled in a single histogram. Fig. 5b shows the response, plotted in the same way, for filters tuned to 0.4 cpd. The shapes of the distributions are similar for first- and second-order filters, being highly peaked around 0. The distributions tend to be more highly peaked for the first-order channel. To allow for a direct comparison across frequencies, Fig. 5c and d shows the distributions plotted as a function of the positional shift that corresponds to each phase disparity using Eq. (8). Fig. 5c shows the responses for the two frequencies for a first-order mechanism, while Fig. 5d shows the results for the second-order mechanism. In both cases, there is a clear relationship between the frequency tuning of the mechanism and the range of responses.

Tanaka and Ohzawa (2006) suggested that the pooling of information across first- and second-order channels might be helpful in the estimation of disparity. The extent to which this pooling might be of benefit depends on the relationship between the energy responses for the two channels. Clearly the second-order channels are most useful if they carry information that first-order channels do not. In this case they would be uncorrelated or have low correlation. On the other hand, second-order channels may provide a corrective function. If the disparity information from the two channels were completely correlated, pooling might be of benefit if the noise in the two channels were to some degree uncorrelated (Oruç, Maloney, & Landy, 2003). Alternatively, since natural images do not conform entirely to the idealised model of disparity, in which the left eye’s view is locally a translation of the right eye’s view, the binocular phase difference for the two channels will not depend purely on the disparity in the centre of the receptive field. For example, multiple different values of disparity will be present across the receptive fields, particularly for the coarsely-tuned second-order channels. We might therefore expect considerable deviation between the phase disparities present in the two channels.

**Fig. 6:**
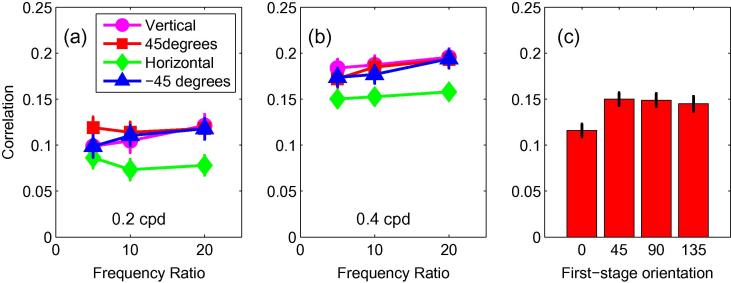
(a) The correlation between the disparities in the first- and second-order channels, for the lower frequency (0.2 cpd) filters. Results show the mean, over all images, of the correlation, calculated separately for each of the 12 combinations of first-stage filter frequency and orientation tuning. (b) The correlations, plotted in the same way, for the higher frequency (0.4 cpd) filters. (c) The mean correlations, over all images and spatial frequencies, as a function of orientation. The correlation was lower when the first-stage filters were horizontal. Error bars show ±1 standard error of the mean.

To establish the degree of similarity between the responses of the channels, we calculated the correlation between the responses of first- and second-order vertically-oriented filters, in which the spatial frequency tuning of the first-order filters was matched to that of the second-stage filtering of the second-order mechanisms. Correlations were calculated separately for each of the 12 carrier frequency/orientation combinations, and for each of the two second-order frequencies. The results are plotted in Fig. 6a and b, which show the mean correlation, over 139 images, in each case. These mean correlations ranged between 0.07 and 0.20. For all 24 comparisons, the mean correlation was significantly greater than zero (smallest *t*(138) = 6.47; *p* < 0.001), with Bonferroni corrections. To determine the effects of the first- and second-stage filtering on these correlations, we also performed a 2 (second-stage filter frequency) × 3 (first-stage filter frequency) × 4 (first-stage filter orientation) repeated measures ANOVA. Correlations were significantly higher when the second-stage frequency was higher (*F*(1,138) = 79.6; *p* < 0.001) and increased with increasing frequency of the first-stage filter (*F*(2,276) = 5.518; *p* = 0.004). Correlations were also affected by the orientation of the first-stage filter (*F*(3,414) = 11.83; *p* < 0.001). Post-hoc pairwise comparisons revealed that this was because the correlation was significantly lower for horizontal first-stage filters than for all other orientations (Fig. 6c). There were no significant interactions.

In order to establish that these correlations were not spurious, we also calculated correlations for phase differences for randomly paired left and right eye images. In all cases, the correlation for correctly matched image pairs was greater than that for randomly matched pairs (smallest *t*(138) = 4.215; *p* < 0.001). Correlations for the randomly-matched images were generally low (the mean value was 0.01) and only two were significant, with Bonferroni corrections. However, even in these cases the magnitudes of the correlations (0.034 and 0.021) were very low.

### 3.3 Disparity estimation from first- and second-order channels

#### 3.3.1 Comparison with first-order filters matched to first-stage frequency tuning

**Fig. 7:**
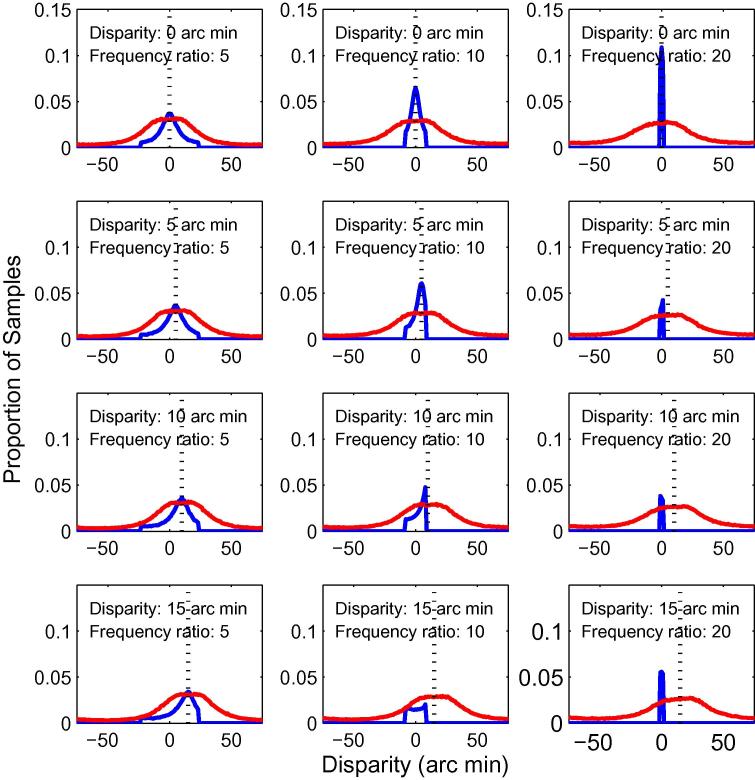
Distributions of winner-takes-all disparity estimates for first- and second-order mechanisms. The first-order results are for filters with the same frequency tuning as the **first-stage** filters of the second-order mechanism. The results for the first-order mechanisms are in blue; the results for the second-order mechanisms in red. The dotted vertical line shows the stimulus disparity. Each row shows the results for a particular stimulus disparity, with the three columns showing results for the three ratios of spatial frequency tuning of the first- and second-stage filters. (For interpretation of the references to colour in this figure legend, the reader is referred to the web version of this article.)

We compared the distributions of disparity estimates between second-order mechanisms, and first-order mechanisms tuned to the same orientation (vertical) and spatial frequency as the first-stage filters. Results are plotted in Fig. 7, for disparities of 0, 5, 10 and 15 arc min. Results are plotted separately for the three spatial-frequency ratios used (5, 10 and 20), for a second-order mechanism tuned to 0.4 cpd; the first-order filters are therefore tuned to 2, 4 or 8 cpd. For zero-disparity, the distributions of disparity estimates are peaked at zero for both first- and second-order mechanisms; the distributions are more sharply peaked for the first-order filters.

As the disparity is increased, the distributions of estimates from the second-order mechanisms continue to be peaked at the correct disparity. However, the first-order estimates become less accurate with increasing disparity, as the disparity becomes greater than the range of their disparity tuning. As expected, this is most evident for the high-frequency filters. Overall, these results show that the second-order channel can increase the range of disparities over that detected by the first-stage filters alone (Wilcox & Hess, 1997).

#### 3.3.2 Comparison with first-order filters matched to second-stage frequency tuning

**Fig. 8:**
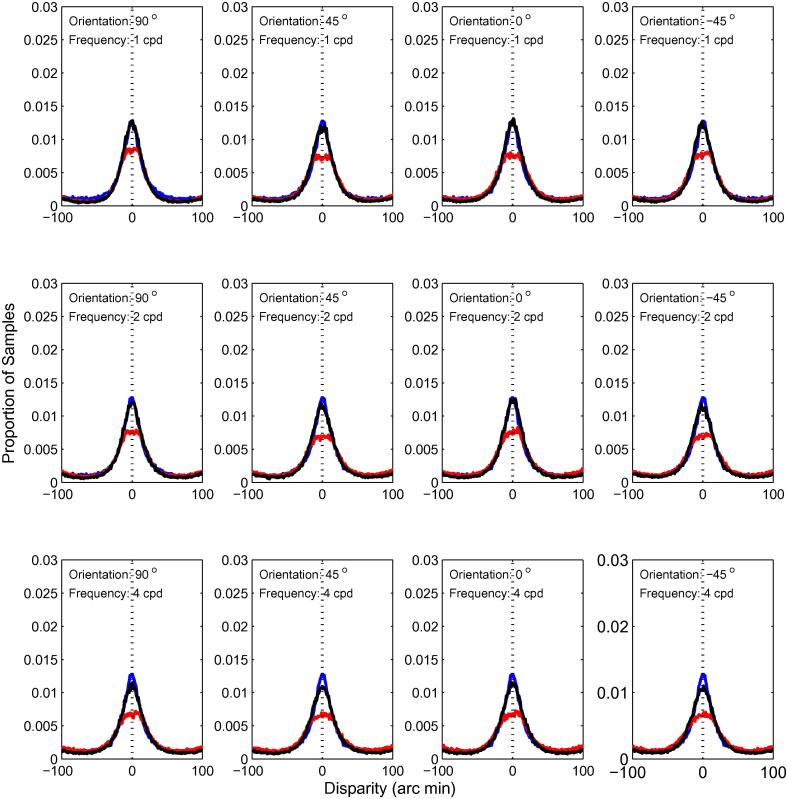
Distributions of winner-takes-all disparity estimates for first- and second-order mechanisms. The first-order results are for filters with the same frequency tuning as the **second-stage** filters of the second-order mechanism. The results for the first-order mechanisms are in blue; the results for the second-order mechanisms in red. The solid black line shows the results after pooling across the two. The dotted black vertical line marks the stimulus disparity (0 arc min). Each column shows the results for a single orientation (indicated in the plots) and each row the results for a single spatial frequency. (For interpretation of the references to colour in this figure legend, the reader is referred to the web version of this article.)

In Fig. 8, we compare the results of the first-order filter, with each of the 12 combinations of frequency and orientation tuning for the first-stage filters. We plotted distributions of disparity estimates in each case for the first- and second-order mechanisms, and for responses pooled across the two. In the latter case, we normalised the population energy response separately for the first- and second-order mechanisms, by dividing each by its maximum response, then added the two before identifying the disparity at which the largest response occurred. This normalisation was necessary to take account of the different magnitudes of responses in the two channels. Results are shown for a disparity of zero. The pattern of results is very similar for all 12 filters. Firstly, the responses for the first- and second-order mechanisms peak at the correct disparity. Secondly, the responses for the first-order filters are more highly peaked than those for either the second-order filters or the pooled responses. These results show that, while second-order responses could potentially contribute to the estimation of disparity, a simple pooling of responses, similar to that performed by individual extra-striate neurons, does not improve the estimation of disparity.

**Fig. 9:**
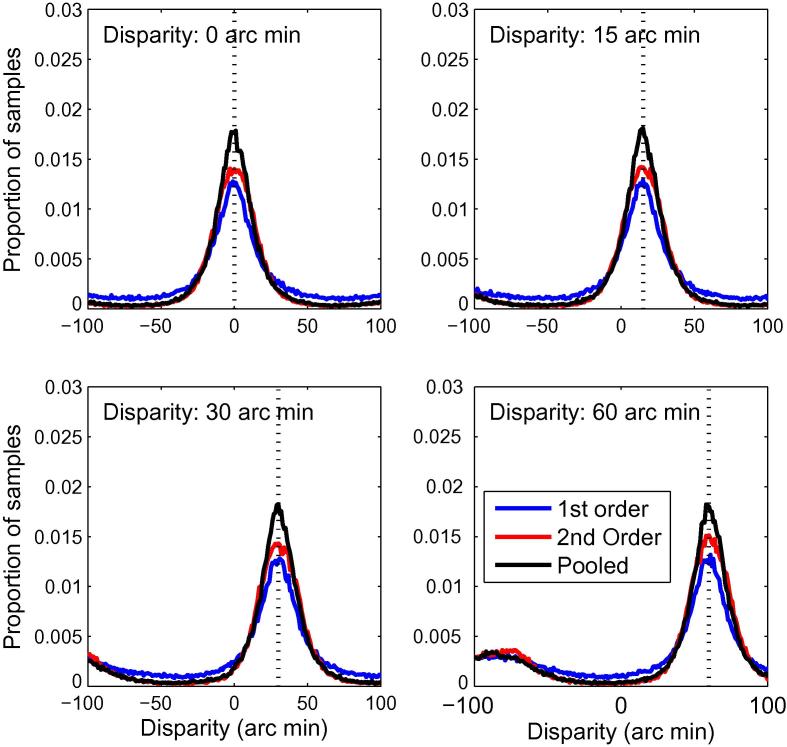
Distributions of winner-takes-all disparity estimates for first-and second-order mechanisms, in which results are pooled over all first-stage filter orientations and frequencies for the second-stage mechanisms. The results for the first-order mechanisms are in blue; the results for the second-order mechanisms in red. The solid black line shows the results after pooling across the two. The dotted black vertical line marks the stimulus disparity. The four plots show results for four stimulus disparities, as labelled. (For interpretation of the references to colour in this figure legend, the reader is referred to the web version of this article.)

However, we performed a second analysis in which the responses of all 12 second-order mechanisms were pooled before the estimation of disparity. These results are shown in Fig. 9. In this case, the responses of the second-order mechanisms are more highly peaked than those of the first-order mechanisms, and the responses pooled across both channels are the most highly peaked of all. These results show that the spatial pooling performed by second-order mechanisms, when combined with a pooling across frequency and orientation, can serve to improve the estimation of disparity. This type of pooling has been proposed as a way to improve the accuracy of disparity estimation (Fleet et al., 1996), and as a way to calculate a local image cross-correlation on the basis of the outputs of linear filters (Allenmark, 2011).

Together, these results suggest that the benefits of second-order mechanisms, as discussed by Tanaka and Ohzawa (2006), are evident when they allow the pooling of information, across the range or orientations and frequencies to which the first-stage filters are tuned, in the estimation of disparity at the scale of the second-stage filter.

## 4 Discussion

We calculated the distributions of phase disparities in binocular natural images. For the first-order channel, the distribution was highly peaked around zero. This is consistent with physiological results that have shown that most cortical neurons are tuned to small disparities (Prince et al., 2002), and psychophysical results that have shown that disparity discrimination is much more accurate for small disparities (Badcock & Schor, 1985). Since the range of phase disparities is limited to ±π, this also means that the range of equivalent positional disparities is inversely proportional to the spatial frequency tuning of the filters. This is consistent with the idea of a size-disparity correlation in disparity estimation (Allenmark, 2011, McKee and Verghese, 2002, Prince et al., 2002, Smallman and MacLeod, 1994, Tsirlin and Allison, 2008, Tyler, 1973, Tyler, 1974, Tyler, 1975).

These results may also be compared to assumptions about the distribution of binocular disparity that have been included in computational models. Read (2002) suggested a Bayesian prior that was similar to, but flatter than, a Gaussian centred at zero. While our analysis also produced a distribution that was centred at zero, it was much more peaked than a Gaussian, rather than flatter. A very similar shape of weighting function, taking the form of an exponential decay, was proposed by Prince and Eagle (2000) in their model of binocular stereopsis. A highly peaked distribution was also found by Hibbard (2007) in a simple occlusion model of the 3D environment, and in calculations based on range data acquired in natural scenes (Liu, Bovik, & Cormack, 2008). The current analysis shows that this provides a good model of the disparity information found in binocular images. Recent empirical data (Sprague, Cooper, Tošić, & Banks, 2015) show that the distribution of disparities, once the fixations of the observer while performing everyday tasks are taken into account, are much more peaked than these theoretical analyses predicted.

The distribution for the second-order channel was similar to, but slightly broader than, that for the first-order channel, when we compared channels with similar spatial-frequency tuning. While extensive data on the distributions of disparity tuning for second-order binocular cells do not exist, it appears that, for cells that respond to both first- and second-order stimuli, the preferred disparity for each type of stimulus is the same (Tanaka & Ohzawa, 2006).

We also assessed the contribution that can be made by a second-order channel to the estimation of disparity. We have found small but significant correlations between the responses of first- and second-order channels, suggesting that second-order channels could have a role in error correction. However this conclusion rests on the assumption that errors in the second-order channel are decorrelated with respect to the first. The remaining variance in the first- and second-order channels, unaccounted for by a linear correlation, could indicate that the second-order channel is providing information that supplements the contribution to disparity estimation made by the first-order channel.

One factor that will affect the second-order information available in the visual system is the effects of local compressive non-linearities that occur early in processing (He and Macleod, 1998, MacLeod et al., 1992). These non-linearities can create first-order components at the orientation and frequency of second-order structure in the original image (Scott-Samuel and Georgeson, 1999, Smith and Ledgeway, 1997). In our analyses, we used the luminance channel of images that were represented as CIE LAB values. This representation includes a compressive nonlinear transformation of raw luminance values. These compressive non-linearities could therefore create additional first-order components, at the frequency and orientation of existing second-order components, in the same way as might occur in the visual system. It is important therefore to consider the effects of these nonlinearities, not just in terms of the effects that a particular image format has on the analysis, but also to understand the effect that early nonlinearities in the visual system may have on the relationship between first- and second-order information in images. To determine the importance of this representation, the analysis was repeated using images in which the luminance was linearised. The results of the analysis are presented in the Supplementary materials. We found that the overall pattern of results was not affected by this transformation. Our conclusions are not therefore critically dependent on the presence of early non-linearities in visual processing.

We found that the second-order channel has the potential to improve the accuracy of disparity estimation both by increasing the range over which this estimation can be performed (Wilcox & Allison, 2009), and by providing a mechanism through which to pool information across space, frequency and orientation in filters that are tuned to detect horizontal disparity (Allenmark, 2011, Fleet et al., 1996).

## Appendix A Supplementary data

Supplementary data 1 This pdf file contains Supplementary “Figs. S1–S5”.
