# Supplementary material for: Encoding and estimation of first- and second-order binocular disparity in natural images
Source: Vision Res. 2016 Mar;120:108–20. doi: 10.1016/j.visres.2015.10.016 (PMC4802249; doi:10.1016/j.visres.2015.10.016)
Supplement: Supplementary data 1 — This pdf file contains Supplementary “Figs. S1–S5”. [file mmc1.pdf]

Encoding and estimation of first- and second-order binocular disparity in natural images:
Supplementary Analysis: The effects of early non-linearities

Paul B. Hibbard, Ross Goutcher and David W Hunter

To determine the effect of the compressive (cube-root) transformation of luminance information inherent in the CIE LAB colour space, we repeated the analysis using the cube of the LAB luminance channel as input. The results of this analysis are presented here, in the same format as the original analysis.

Distributions of phase disparities

Figure S1 shows the distributions of phase disparities in the responses of the Gabor filters, plotted in the same way as Figure 5. These distributions were not substantially affected by the image transformation.

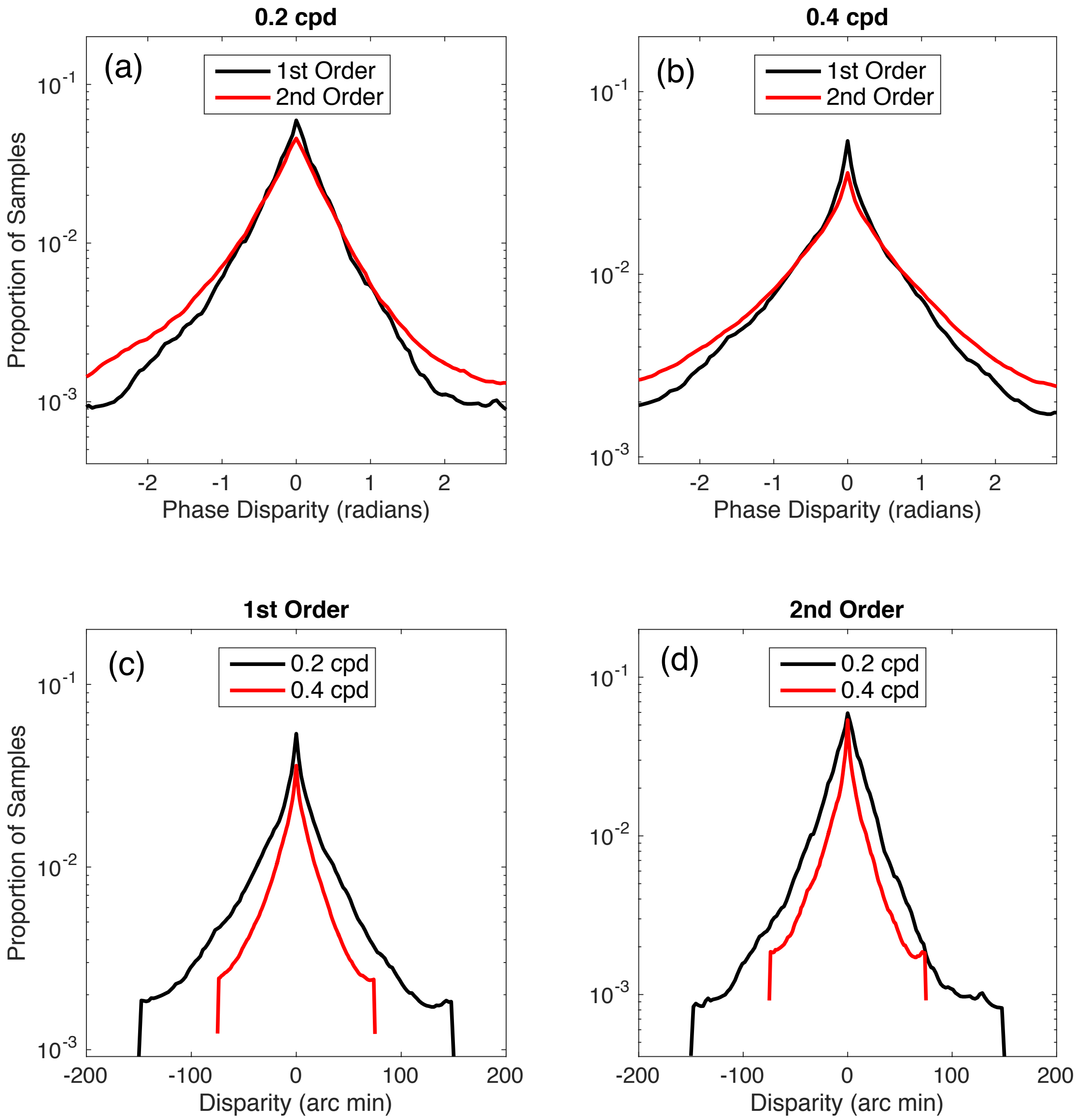

Figure S1 *Phase distributions with linearised input luminance values. Data are plotted as in figure 5.*

Correlations between the responses of first- and second-order channels for the transformed luminance data are plotted in Figure S2. These data are plotted in the same way as in Figure 6, and were analysed in the same way. For all 24 comparisons, the mean correlation was significantly greater than zero (smallest $t(138)= 9.00$; $p<0.001$), with Bonferroni corrections. To determine the effects of the first- and second-stage filtering on these correlations, we also performed a 2 (second-stage filter frequency) x 3 (first-stage filter frequency) x 4 (first-stage filter orientation) repeated measures ANOVA. Correlations were significantly higher when the second stage frequency was higher ($F(1,138)=29.6$; $p<0.001$) and increased with increasing frequency of the first-stage filter ($F(2,276)=28.87$; $p<0.001$). Correlations were also affected by the orientation of the first-stage filter ($F(3,414)=2.758$; $p<0.042$). Post-hoc pairwise comparisons revealed that this was because the correlation was significantly lower for horizontal first-stage filters than for all other orientations. There were no significant interactions.

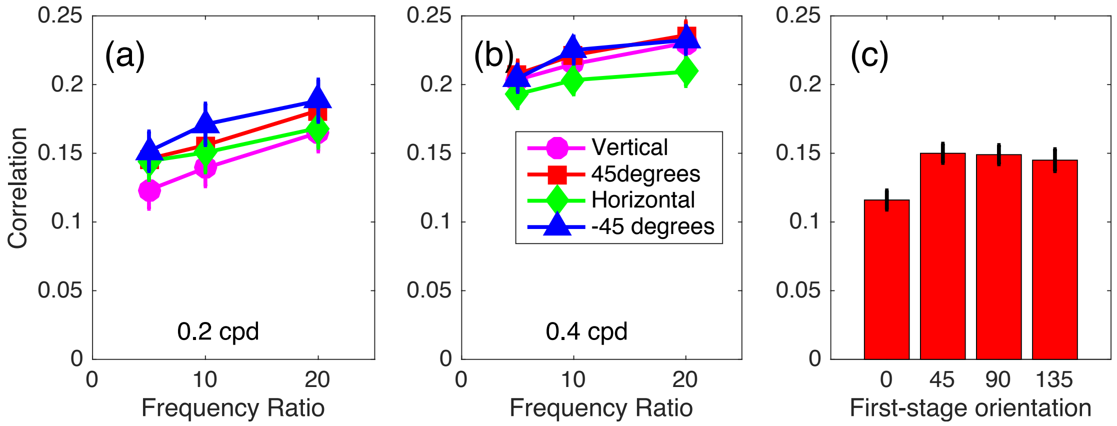

Figure S2 *Correlations between the responses of first- and second-order channels, with linearised input luminance values. Data are plotted as in Figure 6.*

Disparity estimation from first- and second-order channels

The estimates of disparity from first- and second-order mechanisms were also recalculated for stimuli in which the luminance values had been linearised. There are plotted in figures S3-S5. The patterns of results do not differ from those plotted in figures 7-9.

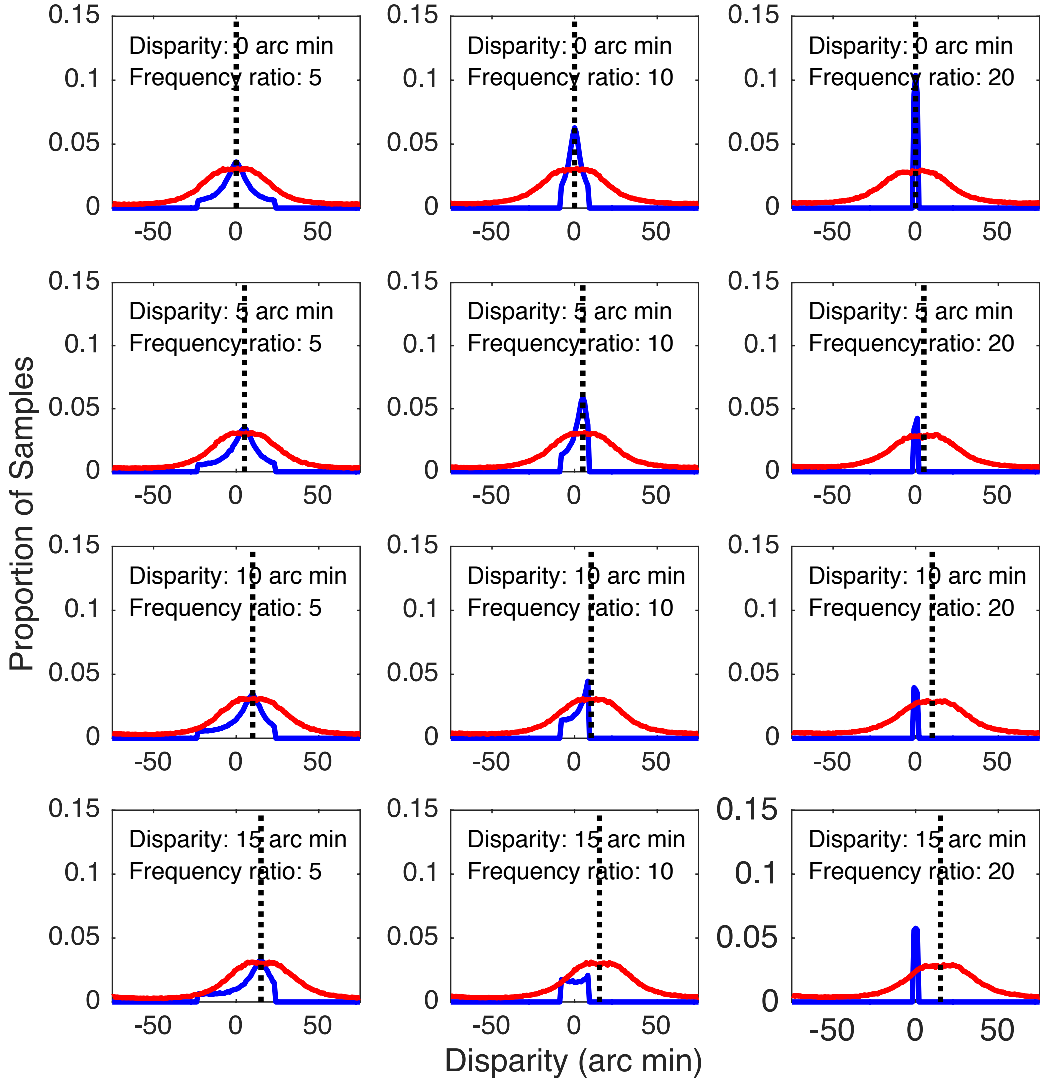

Figure S3 *Distributions of disparity estimates for linearised input images.* The first-order results are for filters with the same frequency tuning as the **first-stage** filters of the second-order mechanism. *Data are plotted in the same was as in figure 7. The results for the first-order mechanisms* are in blue; the results for the second-order mechanisms in red.

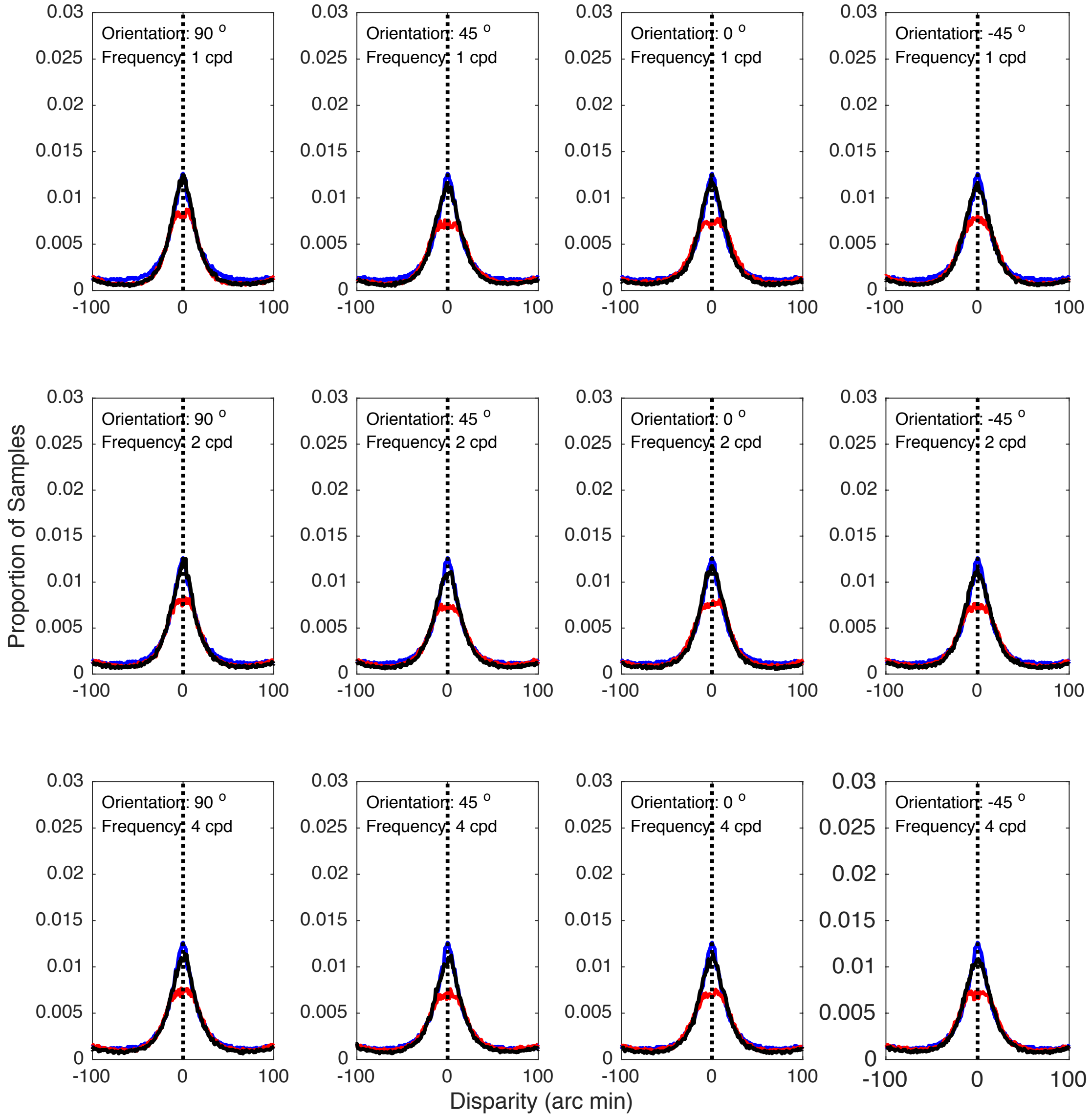

Figure S4 *Distributions of disparity estimates for linearised input images.* The first-order results are for filters with the same frequency tuning as the **second-stage** filters of the second-order mechanism. Data are plotted as in figure 8. The results for the first-order mechanisms are in blue; the results for the second-order mechanisms in red. The solid black line shows the results after pooling across the two. The dotted black vertical line marks the stimulus disparity (0 arc min). Each column shows the results for a single orientation (indicated in the plots) and each row the results for a single spatial frequency.

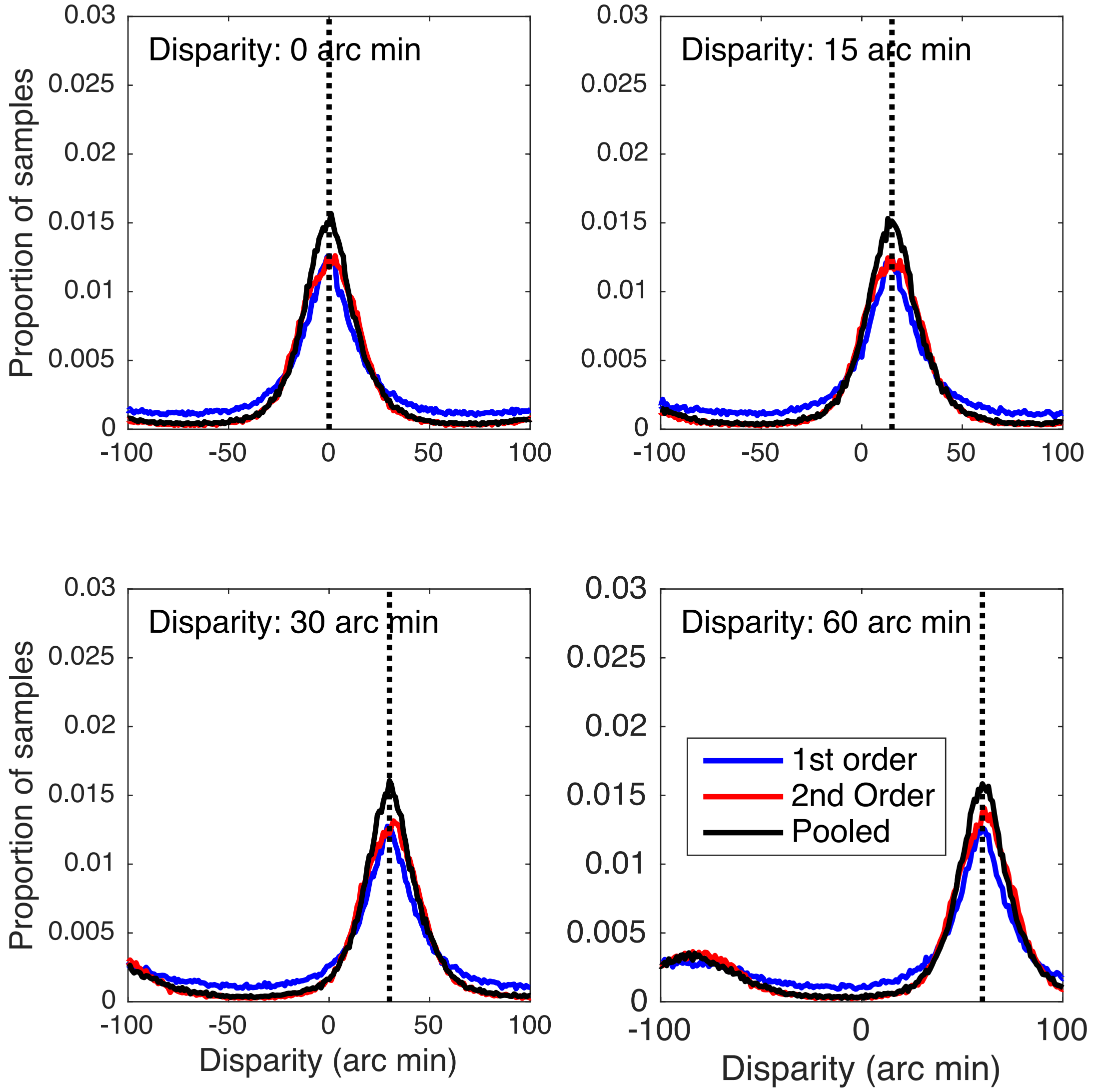

Figure S5 Disparity estimates with pooling over orientation and spatial frequency. Data are plotted as in figure 9. The results for the first-order mechanisms are in blue; the results for the second-order mechanisms in red. The solid black line shows the results after pooling across the two. The dotted black vertical line marks the stimulus disparity. The four plots show results for four stimulus disparities, as labelled.
